# Smart Device–Based Therapy on Hand Motor Function Improvement in Stroke Survivors During Rehabilitation: Scoping Review

**DOI:** 10.2196/73533

**Published:** 2025-11-03

**Authors:** Kristine Krumina, Una Krumina, Agnese Mikelsone, Liva Araka, Klinta Luize Sprudza, Gerda Madara Ziemele, Guna Semjonova

**Affiliations:** 1 Department of Residency, Riga Stradiņš University, Dzirciema Street 16Riga, LV-1007, Latvia, +371 67409125; 2 National Rehabilitation Centre “Vaivari”, Jurmala, Latvia; 3 Faculty of Medicine, Riga Stradiņš University, Riga, Latvia; 4 Department of Rehabilitation, Faculty of Health and Sports Sciences, Riga Stradiņš University, Riga, Latvia

**Keywords:** stroke, smart devices, smart device–based therapy, telerehabilitation, hand motor function

## Abstract

**Background:**

The prevalence of upper limb impairment ranges from 40% to 50% in the chronic phase of stroke, presenting a significant public health challenge. Although traditional therapy effectively improves hand motor function, it often faces accessibility challenges. Telerehabilitation, particularly smart device–based therapy, provides a scalable and engaging alternative, although its effectiveness still requires further investigation.

**Objective:**

This study aimed to identify smart device–based therapy interventions for improving hand motor function in stroke survivors during rehabilitation and to assess their effectiveness in hand motor function improvement in comparison with traditional therapy methods.

**Methods:**

A scoping review was conducted following the PRISMA-ScR (Preferred Reporting Items for Systematic Reviews and Meta-Analyses Extension for Scoping Reviews) guidelines to identify and evaluate smart device–based therapy interventions aimed at improving hand motor function in stroke survivors. A comprehensive literature search was performed across multiple databases, including Web of Science, PubMed (MEDLINE), EBSCO Complete, Google Scholar, Science Direct, ClinicalKey, and Scopus. Studies were screened based on predefined inclusion criteria, focusing on clinical trials that investigated the effectiveness of smart device–based rehabilitation approaches. Data extraction was carried out systematically, capturing key study characteristics, intervention types, and outcome measures. The effectiveness of these interventions was assessed and synthesized to provide a comprehensive overview of their impact on hand motor function recovery in stroke rehabilitation.

**Results:**

A total of 958 studies were identified, of which 17 met the inclusion criteria. The studies encompassed various research designs, including randomized controlled trials (n=9), pilot feasibility studies (n=3), and a comparative nonrandomized trial (n=1). The interventions used diverse digital strategies, including gamified rehabilitation programs (n=14), virtual or mixed reality systems (n=7), Kinect camera or sensor-based approaches (n=6), Jintronix software platforms (n=4), robot-assisted devices (n=3), and tablet- or app-based rehabilitation systems (n=7). Overall, smart device–based therapies were associated with improvements in hand motor function, frequently reaching clinically meaningful thresholds, while also enhancing patient engagement and adherence.

**Conclusions:**

The findings of this scoping study highlight the significant potential of smart device therapies for enhancing hand motor functions among stroke survivors. The development of smart devices is an evolving process, highlighting the need for future studies to assess their long-term effectiveness, optimize intervention designs, and explore their broader application in stroke rehabilitation.

## Introduction

### Background

Stroke remains a significant global health challenge, ranking as the second leading cause of death and the third leading cause of death and disability combined worldwide. The 2022 Global Stroke Factsheet reveals a 50% increase in the lifetime risk of stroke over the past 17 years, with 1 in 4 individuals now facing this threat [1]. The prevalence of upper limb impairment ranges from 50% to 80% in the acute phase to 40% to 50% in the chronic phase of stroke [2]. This prevalence imposes significant demands on health care systems, with rehabilitation needs far outpacing available resources, as noted by the World Health Organization [3].

Traditional therapy for hand motor recovery is highly effective, offering personalized guidance, feedback, and support for both gross and fine motor deficits. However, it is often inaccessible for patients in remote areas, costly, time-consuming, and constrained by a shortage of professionals. In addition, its repetitive nature can reduce patient motivation and adherence, underscoring the need for more engaging and scalable solutions [4].

Telerehabilitation has emerged as a promising solution, offering scalable and accessible interventions to bridge the gap between demand and supply, especially accelerated by the COVID-19 pandemic, which has catalyzed a rapid technological transformation in post–stroke rehabilitation, driving the adoption of telerehabilitation services delivered directly to the homes of stroke survivors [5]. Among these innovations, smart device–based therapy holds the promise of improving hand motor function through gamified, interactive designs that enhance engagement and adherence [6]. Smart devices, such as wearables, mobile apps, and sensor-integrated equipment, provide real-time feedback, track patient progress, and enable therapists to monitor performance remotely. Such systems often integrated gamification and virtual reality elements to improve engagement and adherence. Studies suggested that these interventions not only enhanced motor outcomes but also positively influenced psychological well-being, such as motivation and satisfaction with therapy [7].

Although there are limited data on the comparative effectiveness of smart device–based therapies, their potential in stroke rehabilitation is promising [8].

### Objective

This study aimed to identify smart device–based therapy interventions for improving hand motor function in stroke survivors during rehabilitation and to assess their effectiveness in comparison with traditional therapy methods. Specifically, the study sought to identify and categorize the types of digital hand rehabilitation devices available to stroke patients, including their functionalities and applications; and to compare the effectiveness of remote smart device–based therapies with traditional rehabilitation methods in enhancing hand motor function in stroke survivors.

### Research Question

The research questions are as follows: What types of digital hand rehabilitation devices are available for remote use by patients with stroke, and how do their functionalities and effectiveness in improving motor function compare with traditional therapy?

## Methods

### Overview

The scoping review was performed according to the Joanna Briggs Institute methodology for scoping reviews [9,10] and was reported following the PRISMA-ScR (Preferred Reporting Items for Systematic Reviews and Meta-Analyses Extension for Scoping Reviews; Checklist 1 ) guidelines [11].

The review was registered on the Open Science Framework [12] to promote transparency, enhance reproducibility, and ensure open access to the data, methods, and findings for the broader scientific community.

### Search Strategy

This scoping review was conducted to identify smart device–based therapy interventions in clinical trials across 7 databases. The databases searched included Web of Science, MEDLINE (via PubMed), EBSCO Complete, ScienceDirect, and Scopus. In addition, literature was retrieved using the search engine Google Scholar and the publisher platform ClinicalKey. The initial search was conducted in December 2023 for studies published between 2018 and 2023. An updated search was performed on August 10, 2025, extending the coverage to studies published from 2018 to 2025. Additional eligible studies were hand-searched. The available literature was screened to identify smart device–based therapy interventions used to improve hand motor function in stroke survivors during rehabilitation, and their levels of evidence were analyzed.

The search used the following primary keywords and corresponding Medical Subject Headings terms: “Stroke,” “Telerehabilitation,” “Smart devices,” and “Upper extremity.” The primary keywords and corresponding Medical Subject Headings terms are listed in Table 1. The search strategy was tailored to align with the specific indexing and search functionalities of each database. Adjustments were made not only to the keywords but also to the search parameters unique to each platform to ensure comprehensive retrieval of relevant studies. The full search strategy for each database is presented in Multimedia Appendix 1.

**Table 1.** Primary keywords and their corresponding MeSH^a^ terms used in database search.

Keyword categories	Keywords
1	“Stroke” OR “Cerebrovascular Accident” OR “CVA”
2	“Telerehabilitation” OR “Tele-rehabilitation” OR “Tele rehabilitation” OR “Remote Rehabilitation” OR “Virtual Rehabilitation” OR “telemedicine” OR “telehealth”
3	“Smart devices” OR “technology” OR “VR” OR “virtual reality”
4	“Hand” OR “upper extremity” OR “upper limb”
Search strategy	((1) AND (2) AND (3) AND (4))

a MeSH: Medical Subject Headings.

### Study Selection Criteria

The inclusion and exclusion criteria are shown in Textbox 1.

Textbox 1. **Inclusion and exclusion criteria**

Inclusion criteria for study selection were as follows:

- Population: adult patients diagnosed with stroke
- Condition: upper limb disability or paresis
- Intervention: technology-based interventions (eg, smart devices) used for rehabilitation
- Setting: experimental group—home-based therapy with or without remote supervision or inpatient treatment without substantial assistance involving smart wearable devices, smartphones, tablets, robotic devices, gamified rehabilitation systems, and other similar technologies
- Outcomes: studies reporting on hand function improvement and rehabilitation progress
- Time frame: papers published between 2018 and August 2025
- Language: studies published in English
- Publication type: peer-reviewed papers, reviews, clinical trials, case studies, systematic reviews, and gray literature
- Availability: full-text papers only

Exclusion criteria for study selection were as follows:

- Population: studies involving pediatric patients
- Setting: inpatient treatment settings that required continuous therapist assistance to set up, guide, or complete the intervention
- Diagnosis: patients with diagnoses other than stroke
- Focus: studies unrelated to hand rehabilitation (eg, lower limb rehabilitation, cognitive rehabilitation without a hand-related component)

### Selection of Sources of Evidence

All identified references were managed using Mendeley (v 1.19.8; Elsevier), and duplicate records were removed using Covidence systematic review software (Veritas Health Innovation). The study selection process followed a structured multistage approach. First, 2 independent reviewers conducted an initial screening of titles and abstracts to identify potentially relevant studies. Studies deemed ineligible based on predefined inclusion and exclusion criteria were excluded at this stage. Full-text screening was performed on the remaining studies by 2 independent reviewers to determine final eligibility. Any disagreements regarding study inclusion were resolved through discussion, and if necessary, a third reviewer provided adjudication. Covidence systematic review software was used throughout the selection process to ensure accuracy and consistency.

### Data Charting and Extraction

A structured data charting framework was developed in Covidence to ensure a systematic and organized approach to data extraction. Two independent reviewers extracted key information from the selected studies, including study characteristics (eg, author, publication year, and country), participant details (eg, sample size, stroke type, and rehabilitation setting), intervention descriptions (eg, technology type and therapy mode), and reported outcomes (eg, functional improvements). To maintain consistency and accuracy, the reviewers engaged in discussions to resolve any discrepancies in the extracted data. If disagreements persisted, a third reviewer provided adjudication. The finalized data were compiled into Microsoft Excel for further synthesis and analysis, enabling a comprehensive comparison of intervention effectiveness across studies.

### Data Items

For each included study, key data points were systematically extracted to facilitate a comprehensive analysis. The extracted data included the study details, study design, participant characteristics, intervention details, outcome measures, and the effectiveness of interventions.

#### Study Details

This included the author(s), publication year, title, and country of origin.

#### Study Design

This included the type of study (eg, randomized controlled trial and pilot study), total number of participants, and patient care setting (eg, home-based, outpatient, and inpatient).

#### Participant Characteristics

This included stroke classification (chronic or subacute), inclusion criteria, and any cognitive assessments performed

#### Intervention Details

This included the type of smart device–based therapy used (eg, virtualand mixed reality, gamification, sensor-based systems, and robotic-assisted rehabilitation), and mode of delivery (asynchronous, synchronous, or hybrid).

#### Outcome Measures

This included the primary and secondary measures assessing hand motor function improvement (eg, Fugl-Meyer Assessment for Upper Extremities [FMA-UE], Stroke Impact Scale [SIS], and Wolf Motor Function Test [WMFT]).

#### Effectiveness of Interventions

These were the findings related to functional recovery and improvements in motor skills.

### Data Synthesis

The extracted data were systematically compiled and analyzed to generate a comprehensive overview of the characteristics and findings of the included studies. Data synthesis was carried out in multiple stages to ensure a structured and thorough evaluation. Initially, all relevant data were organized in Microsoft Excel (Microsoft Corporation), where key variables—such as study design, participant demographics, intervention type, outcome measures, and reported effectiveness—were categorized.

Quantitative findings, including the effectiveness of smart device–based therapy on hand motor function improvement, were aggregated based on standardized outcome measures, such as the FMA-UE, SIS, and WMFT. These results were interpreted to determine trends in functional improvements observed among stroke survivors who underwent smart device–based rehabilitation. In addition, variations in intervention designs—such as the use of virtual and mixed reality, gamified rehabilitation platforms, robotic-assisted devices, and wearable sensors—were examined to explore how different technological solutions contribute to rehabilitation outcomes. Consideration was also given to the mode of therapy delivery (asynchronous, synchronous, or hybrid), as well as contextual factors influencing treatment effectiveness, such as study settings (home-based vs inpatient rehabilitation) and patient characteristics (chronic vs subacute stroke).

## Results

### Study Selection

**Figure 1.**
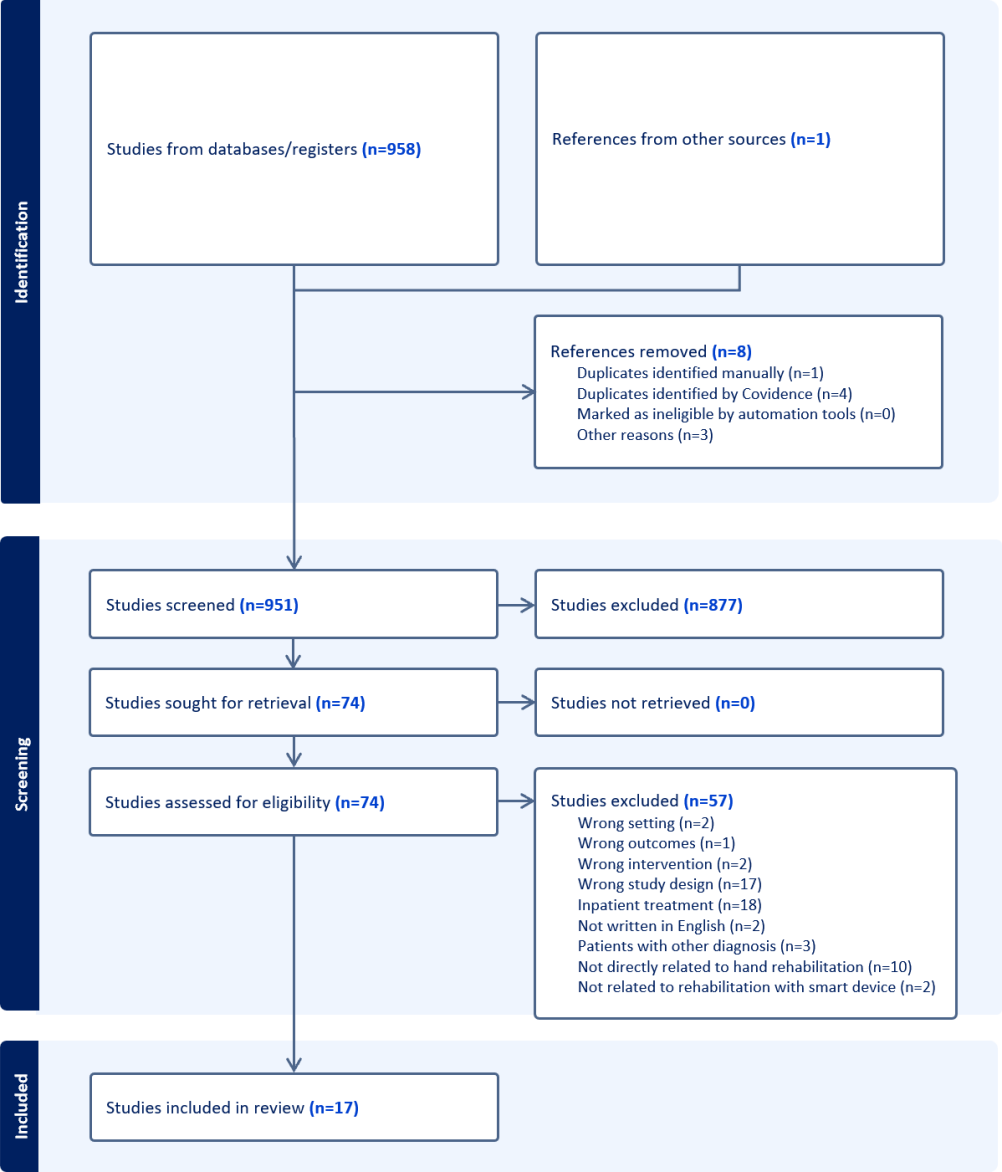
PRISMA (Preferred Reporting Items for Systematic Reviews and Meta-Analyses) flow diagram for the study selection in the scoping review, depicting the identification of studies via databases and registers.

A total of 958 records were identified from databases; after removing duplicates, 951 papers underwent screening, and following a review of titles and abstracts, 877 papers were excluded. Full texts of 74 papers were acquired for further screening. During the full paper text screening, 57 studies were excluded, and the remaining 17 [13-29] studies were selected for extraction. Figure 1 illustrates the process.

Of the 958 identified studies, 17 [13-28] met the inclusion criteria. The studies included randomized controlled trials (RCTs; n=9) [13,16,18-23,27-29], non-RCT (feasibility and experimental; n=4) [15,17,26,27], pilot and feasibility studies (n=3) [14,19,20], and comparative non-RCT (n=1) [24]. The interventions used diverse digital strategies, including gamification (n=14) [13-16,18-26,28], virtual and mixed reality devices (n=7) [14-16,18,22,23,25], Kinect camera and sensor (n=6) [13,15,16,22,23,25], Jintronix software (n=4) [15,16,22,23], robot-assisted devices (n=3) [14,20,24], and app or platform based (n=7) [15,16,22,23,25,26,29]. By stroke phase, most studies included chronic cohorts (n=12), with several spanning subacute populations (n=6); we found no acute-only trials. These studies involved 508 participants. Smart device–based interventions were delivered via asynchronous (n=6) [15,16,22,23,25,29], synchronous (n=1) [17], and hybrid (n=10) [13,14,18-21,24,26-28] approaches. Patient care settings were predominantly home-based (n=14) [13,14,16-20,22,24-29], with several studies also involving outpatient components (n=7) [14,15,17,18,23,24,27] and inpatient settings in 2 studies (n=2) [21,28]. In 3 studies, cognitive tests were used for outcome measures—Montreal Cognitive Assessment [22,27,29] and Neurobehavioral Functioning Inventory [29].

### Study Characteristics and Intervention Effectiveness by Device

An overview of interventions and outcome of each used device of 17 included studies is summarized in Tables2  3.

**Table 2.** Characteristics of included studies.

Author, year	Title	Type of study	Experimental group device type, name	Total participants	Experimental group care setting	Control group interventions in RCT^a^
Adams et al [13], 2023	Telehealth guided virtual reality for recovery of upper extremity function following stroke (GRASP)	RCT	Custom finger sensors+SaeboGlove+GRASP sensor hub (Saebo Inc)+Kinect (Microsoft Corp)	18	Home-based asynchronous HEP^b^+biweekly synchronous telehealth OT^c^	Usual and customary care (no GRASP system)
AguirreOllinger et al [14], 2024	Telerehabilitation using a 2D planar arm rehabilitation robot for hemiparetic stroke: a feasibility study of clinic-to-home exergaming therapy	Prospective pilot feasibility	2D planar end-effector robot (HMan (Articares Pte Ltd), ARTICARES)+home exergaming	12	Clinic onboarding → home (minimally supervised)	—^d^
Allegue et al [15], 2021	Single case evaluation of VirTele (Jintronix+Reacts) for home-based upper limb stroke rehabilitation	Single case study	VirTele^e^ (Jintronix (Jintronix Inc)+Reacts (Technomed))	1	Home-based with telesupervision	—
Allegue et al [16], 2022	VirTele: a patient-teletherapist program combining exergames (Jintronix) and videoconferencing (Reacts) for chronic stroke (feasibility trial)	Randomized feasibility trial	Jintronix+Reacts videoconferencing; motivational interviewing	9	Home-based; synchronous telesessions	GRASP home program
Althaf et al [17], 2024	Effectiveness of mirror therapy through telerehabilitation on upper extremity performance in hemiparetic stroke patients: an experimental study	Experimental study	Mirror box setup via telerehabilitation	60	Home-based	Face-to-face mirror therapy
Ase et al [18], 2025	Effects of home-based virtual reality upper extremity rehabilitation in persons with chronic stroke: a randomized controlled trial	RCT	RAPAEL Smart Glove (Neofect)+VR^f^ software	14	Home program+weekly outpatient OT	Conventional home exercise program+weekly outpatient OT
Bressi et al [19], 2023	Upper limb home-based robotic rehabilitation in chronic stroke patients: a pilot study	Pilot feasibility study	iCONE (Wearable Robotics srl) end effector planar robot with remote supervision	13	Home-based telerehabilitation	—
Germanotta et al [20], 2025	A robotic rehabilitation intervention in a home setting during the COVID19 outbreak: a feasibility pilot study in patients with stroke	Nonrandomized feasibility pilot	Planar robot (iCONE) with telemonitoring and teleconsultation	18	Home-based telerehabilitation	—
Guo et al [21], 2023	A remote rehabilitation training system based on wearable devices for stroke inpatients: randomized clinical trial	RCT	Wearable IMU^g^ based remote training system+app and cloud	109	Inpatient rehabilitation halls (remoteguided)	Conventional PT or OT^h^ without the wearable system
Hernández et al [22], 2022	Feasibility of a virtual reality exergame (Jintronix) versus GRASP for home-based upper extremity stroke rehabilitation	Randomized feasibility trial	Jintronix VR exergaming (Kinect)+remote support	51	Home-based telerehabilitation	GRASP home program
Norouzi Gheidari et al [23], 2020	Supplementary telerehabilitation using Jintronix for outpatients after stroke: pilot randomized trial	Pilot randomized controlled trial	Jintronix VR exergaming platform (Kinect)	18	Outpatient+home telerehabilitation supplement	Standard outpatient rehabilitation without VR supplement
Pavan et al [24], 2024	Effectiveness of two models of telerehabilitation in improving recovery from subacute upper limb disability after stroke: robotic versus non-robotic	Comparative non-RCT	RAPAEL Smart Glove ^i^	30	Home-based telerehabilitation (remote supervision)	Nonrobotic telerehabilitation of equivalent dose
Qiu et al [25], 2020	Development and pilot evaluation of the home-based virtual rehabilitation system (HoVRS) for chronic stroke	Feasibility single-arm	HoVRS^j^ (developed by University of California Irvine; leap motion–based VR exercises)	15	Home-based with remote monitoring	—
Rozevink et al [26], 2021	Validity of the MERLIN arm weight supporting device for telerehabilitation after stroke	Nonrandomized experimental study	MERLIN^k^ (developed by University of Groningen, Netherlands; unactuated arm weight support device)+remote coaching	10	Home-based telerehabilitation context	—
Simpson et al [27], 2024	A randomized control trial of a virtually delivered program for increasing upper limb activity after stroke (VABC)	RCT	Virtual arm boot camp (exercise+TENZR wrist-worn sensor feedback [BioInteractive Technologies]+6 virtual sessions)	73	Fully virtual or home-based	Waitlist or usual care
Toh et al [28], 2024	Effects of a wearable-based intervention on the hemiparetic upper limb in persons with stroke: a randomized controlled trial	RCT	Wrist-worn wearable+reminder app (SRAPP [developed by the Chinese University of Hong Kong]) with remote guidance	40	Home or outpatient telerehabilitation	Usual care without the wearable system
Wilson et al [29], 2021	Home-based (virtual) rehabilitation improves motor and cognitive function for stroke patients: RCT of the Elements (EDNA22) system	RCT	EDNA22 (Dynamic Neural Arts) tablet-based virtual rehabilitation system	17	Home-based virtual rehabilitation	GRASP home exercise program (active control)

a RCT: randomized controlled trial.

b HEP: home exercise program.

c OT: occupational therapy.

d —: not applicable.

e VirTele: virtual reality exergames combined with a telerehabilitation app.

f VR: virtual reality.

g IMU: inertial measurement unit.

h PT: physical therapy.

i TR: telerehabilitation.

j HoVRS: home-based virtual rehabilitation system.

k MERLIN: Home care arm rehabilitation.

**Table 3.** Overview of included studies treatment effect.

Author, year	Aim of study	Outcome measure	Treatment effect
Adams et al [13], 2023	Effectiveness of GRASP glove program	FMA-UE^a^ WMFT-FA^b^	+10.1 (95% CI 6.7‐13.5; *P*<.001); 8.6 units greater versus control (95% CI 4.9‐12.2; *P*<.001) +0.73 (95% CI 0.26‐1.19, *P*=.005)
Aguirre-Ollinger et al [14], 2024	Feasibility of H-Man robotic telerehabilitation	FMA ARAT^c^	Change +3.7 (*P*=.02) Change +4.8 (*P*=.004)
Allegue et al [15], 2021	VirTele^d^ feasibility	FMA-UE SIS^f^ (hand function, ADLs)^g^	Improved above MCID^e^ (4.25‐7.25) Gains exceeded MCID (9.4‐14.1)
Allegue et al [16], 2022	VirTele versus conventional therapy	FMA-UE MAL^h^-30 SIS-16	50% improved 2 participants reached MCID 1 participant exceeded MCID
Althaf et al [17], 2024	Mirror therapy via telerehabilitation	FMA-UE WMFT-FA	No significant difference between groups, *P*=.673 No significant difference between groups, *P=.*396
Ase et al [18], 2025	Home-based VR^i^ RCT^j^	FMA-UE BBT^k^ JTT^l^	Robotic +8.9 (SD 1.9) versus control +1.7 (SD 1.9), *P*=.027 Significant difference between groups, *P*=.014 Significant difference between groups, *P*=.-002
Bressi et al [19], 2023	iCONE robot home-based rehabilitation	FMA-UE MAS^m^ (elbow) Robotic indices	Improved not significant Significant decrease, *P*=.017 Independence *P*=.04, size *P*=.019, and duration *P*=.002 all improved
Germanotta et al [20], 2025	Robotic rehabilitation during COVID—iCONE	FMA-UE	11 of 18 patients improved beyond MCID (5.25)
Guo et al [21], 2023	Wearable-based rehabilitation system	FMA FMA-UE	+17.56 (SD 11.65), significant +11.28 (SD 8.59), significant
Hernández et al [22], 2022	VR serious game versus home exercise	FMA-UE	56% (9/16) reached MCID, *P*=.045
Norouzi-Gheidari et al [23], 2020	VR exergame feasibility	SIS mobility SIS physical	+5.5%, *P*=.017 +6.7%, *P*=.041
Pavan et al [24], 2024	Robotic versus nonrobotic telerehabilitation	FMA-UE ARAT	Robotic +4.44 (SD 0.79) versus nonrobotic +10.79 (SD 2.17) Robotic +3.25 (SD 1.68) versus nonrobotic +7.71 (SD 2.05)
Qiu et al [25], 2020	HoVRS^n^ feasibility	FMA-UE	+5.2 (SE 0.69, 95% CI 3.66‐6.71; *P*<.001); exceeds MCID 4.25
Rozevink et al [26], 2021	MERLIN^o^ training	WMFT^p^ FMA-UE ARAT	+3.8, *P*=.006 Significant (ηp²=0.60) Significant (ηp²=0.27)
Simpson et al [27], 2024	Virtually delivered UL^q^ program	Wearable activity counts	MD=368, *P*=.046
Toh et al [28], 2024	Wearable-based RCT	FMA-UE	MD=2.05, *P*=.036
Wilson et al. [29], 2021	Compare EDNA^r^ system with active control for motor, cognitive, and functional outcomes	BBT 9HPT^s^ MoCA^t^	Large effect, g=0.90 Moderate effect, g=0.55 Moderate-large effect, g=0.70

a FMA-UE: Fugl-Meyer Assessment for Upper Extremities.

b WMFT-FA; Wolf Motor Function Test Functional Ability.

c ARAT: Action Research Arm Test.

d VirTele: virtual reality exergames combined with a telerehabilitation app.

e MCID: minimal clinically important difference.

f SIS: Stroke Impact Scale.

g ADL: activities of daily living.

h MAL-30: Motor Activity Log-30.

i VR: virtual reality.

j RCT: randomized controlled trial.

k BBT: Box and Block Test.

l JTT: Jebsen-Taylor Hand Function Test.

m MAS: Modified Ashworth Scale.

n HoVRS: home-based virtual rehabilitation system.

o MERLIN: HoMEcare aRm rehabiLItatioN.

p WMFT: Wolf Motor Function Test.

q UL: upper limb.

r EDNA: Elements by Dynamic Neural Art.

s 9HPT: 9-Hole Pegboard Test.

t MoCA: Montreal Cognitive Assessment.

## Discussion

### Principal Findings

The scoping review highlighted the potential of smart device–based therapies in improving hand motor function among stroke survivors. The review synthesized evidence from 17 studies and identified 5 main types of digital hand rehabilitation devices: virtual and mixed reality systems [15,16,18,22,23,25], which provide immersive environments for interactive training; wearable sensor-based devices, such as motion-tracking gloves [13] and inertial measurement unit–based sensors [21,27,28], enabling real-time feedback and remote monitoring; robotic-assisted rehabilitation devices [14,19,20,24,26], which deliver mechanical support and facilitate guided movement practice; tablet- or app-based rehabilitation platforms [13,15,16,22,23,25,29], offering structured exercise programs with interactive and gamified elements; camera- or Kinect-based motion tracking systems [13,15,16,22,23,25], allowing gesture-based therapy without direct device contact; and mirror therapy device [17], representing a low-tech but adaptable telerehabilitation tool. These smart technologies enhance rehabilitation and can complement or even surpass traditional rehabilitation methods in certain contexts. In line with our findings, several recent trials have further strengthened the evidence on smart device–based and telerehabilitation approaches for upper limb recovery after stroke. For example, Pavan et al [24] demonstrated that both robotic and nonrobotic telerehabilitation models significantly improved motor and cognitive skills in subacute stroke patients, highlighting the feasibility of integrating such solutions into home-based care. Similarly, Simpson et al [27] reported that a virtually delivered program combined with a wearable device increased upper limb activity, emphasizing the role of remote therapist support and objective monitoring. Toh et al [28] further confirmed the efficacy of wearable-based interventions, where a novel *Smart Reminder* device improved upper limb function and adherence in a randomized controlled trial. Taken together, these results reinforce the promise of smart device–based rehabilitation and provide an important context for examining how such approaches compare directly with established traditional therapies.

### Comparison With Traditional Therapy

Treatment effectiveness was compared with a variety of conventional or control interventions (hereafter collectively referred to as *traditional therapy*) in 11 studies [13,16-18,21-24,27-29]. These comparators were heterogeneous and included usual care, GRASP home programs, face-to-face mirror therapy, and conventional home exercise programs with outpatient OT or PT. FMA-UE improved across studies, with gains ranging from 6.8 to 17.56 points in experimental groups, frequently exceeding the minimal clinically important difference and indicating clinically meaningful motor recovery. Functional metrics, such as WMFT, Box and Block Test, and Action Research Arm Test, showed moderate to large improvements, with effect sizes ranging from *g*=0.55 to *g*=0.90. Cognitive outcomes were less frequently assessed, but in the few studies that did, the Montreal Cognitive Assessment demonstrated a moderate-to-large effect [22,27,29].

### Limitations and Challenges Identified in the Studies

#### Our Study Limitations

This review has a few limitations. In this study, only studies in English were reviewed and included, which may have restricted the scope of the findings. The limited number of studies highlighted the potential need to reconsider the exclusion criteria. For instance, our choice to focus solely on remote interventions may have further narrowed the pool of eligible studies. Another limitation is the exclusion of interventions delivered in inpatient settings with substantial assistance. While this approach allowed us to focus on independently usable technologies in home or community environments, it may have reduced the applicability of our findings to individuals with more severe impairments who typically require support during training. As a result, certain devices that demonstrate effectiveness only when used with clinical assistance may be underrepresented in this review. Moreover, inconsistent methodologies and outcome measures across studies hindered direct comparisons, making it challenging to draw robust conclusions from user experience data. In our research, we did not include patient experiences with technologies or usability aspects, as that was not our aim, and these were primarily addressed in separate studies. However, user satisfaction and engagement play a crucial role in enhancing therapy effectiveness [16], highlighting the importance of considering these factors in future research. Moreover, rapid advances in smart technology suggest that findings may require updates as new devices emerge. Several promising smart rehabilitation tools are currently under development, although many remain in early stages, with limited evidence on their clinical efficacy and usability [30].

#### Limitations of the Included Studies

There were several limitations related to the included studies. First, the included studies differentiated stroke cases by the timing of the event but did not distinguish between ischemic and hemorrhagic strokes. These types vary in recovery mechanisms, potentially affecting device efficacy [31]. Second, in our study, only 1 included cognitive test for outcome measures. Given that cognitive impairments—such as deficits in memory, attention, and executive function—are common among stroke survivors and impact hand motor function recovery and rehabilitation success [32]. Third, the included studies used various outcome measures to assess the effectiveness of the interventions, such as FMA-UE, MAL, SIS, and Box and Block Test, which made it difficult to make direct comparisons.

### Implications for Clinical Practice and Rehabilitation Programs

Although the included studies varied in design and sample size, the overall findings indicate that smart device–based interventions are both safe and feasible for telerehabilitation, either delivered independently or in combination with conventional therapy. Several systems already integrated into clinical practice, such as Jintronix [15,16,22,23], have demonstrated that commercial platforms can be effectively adapted for home-based programs, enhancing patient motivation and adherence. Other tools, including the home-based virtual rehabilitation system [25] and wearable sensor-based devices [21,27,28], showed potential to support partially autonomous rehabilitation at home by providing real-time feedback, objective monitoring, and remote therapist supervision. Robotic-assisted devices such as H-Man and iCONE [14,19,20,24] offered mechanical support for patients with more severe impairments and achieved clinically meaningful improvements even with limited therapist input.

### Future Research Directions

The findings of this scoping review highlight the significant potential of smart device–based therapy for enhancing hand motor function among stroke survivors. Building on these promising outcomes, several future research directions can help advance the development, implementation, and effectiveness of these interventions. Future research should explore the integration of cognitive and hand motor function rehabilitation to address the multifaceted needs of stroke survivors. Developing comprehensive standardized rehabilitation protocols that include interventions targeting memory, attention, and executive hand function alongside motor recovery could provide a more holistic approach to therapy. In addition, the absence of a single standardized outcome measure for hand motor recovery remains a major limitation of the current evidence base, introducing variability and potential bias across studies. Future work should therefore prioritize the development and adoption of consensus-based, standardized outcome measures to allow more reliable comparison and synthesis of findings across trials.

Emerging innovations in augmented reality and artificial intelligence present exciting opportunities for advancing stroke rehabilitation [33]. Augmented reality can provide immersive, engaging therapy environments, whereas artificial intelligence–driven systems have the potential to deliver adaptive, personalized rehabilitation. In addition, research on more sophisticated sensor and feedback mechanisms can refine therapy delivery and enhance patient outcomes [34], which leads to the crucial future recommendation that technology and innovations should focus on creating user-friendly devices and protocols that empower patients to engage in effective, autonomous rehabilitation from the comfort of their homes, addressing the growing demand for accessible care.

### Conclusions

Smart device–based interventions demonstrate possibilities in enhancing hand motor function and overall rehabilitation outcomes in stroke survivors. These results strongly advocate for the integration of smart devices into standard rehabilitation protocols to maximize accessibility and improve patient-specific outcomes. Further research and development are warranted to expand the use of smart technologies, optimize their effectiveness, and integrate them more broadly into stroke rehabilitation practices.

## Supplementary material

10.2196/73533 Multimedia Appendix 1 Search strategy.

10.2196/73533 Checklist 1 PRISMA-ScR (Preferred Reporting Items for Systematic Reviews and Meta-Analyses Extension for Scoping Reviews) fillable checklist.

## Abbreviations

FMA: Fugl-Meyer assessment
FMA-UE: Fugl-Meyer assessment for upper extremities
PRISMA: Preferred Reporting Items for Systematic Reviews and Meta-Analyses
PRISMA-ScR: Preferred Reporting Items for Systematic Reviews and Meta-Analyses Extension for Scoping Reviews
SIS: Stroke Impact Scale
WMFT: Wolf Motor Function Test
