# Supplementary material for: Smart Device–Based Therapy on Hand Motor Function Improvement in Stroke Survivors During Rehabilitation: Scoping Review
Source: JMIR Rehabil Assist Technol. 2025 Nov 3;12:e73533. doi: 10.2196/73533 (PMC12587065; doi:10.2196/73533)
Supplement: Multimedia Appendix 1 [file rehab-v12-e73533-s001.docx]

**Appendix 2**: Database search details.

Database limit: 2018 -2025, english, adult

| **Database** | **Search terms** | **Number of studies** |
| --- | --- | --- |
| ClinicalKey | ((""Stroke"" OR ""Cerebrovascular Accident"" OR ""CVA (Cerebrovascular Accident)"" ) AND (""Telerehabilitations"" OR ""Tele-rehabilitation"" OR ""Tele rehabilitation"" OR ""Remote Rehabilitation"" OR ""Virtual Rehabilitation"" OR ""telemedicine"" OR ""telehealth"") AND (""smart devices"" OR ""device"" OR ""technology"" OR ""VR"" OR ""virtual reality"") AND (""hand"" OR ""upper extremity"" OR ""upper limb"")) | 495 |
| EBSCO complete | ((""Stroke"" OR ""Cerebrovascular Accident"" OR ""CVA (Cerebrovascular Accident)"") AND (""Telerehabilitations"" OR ""Tele-rehabilitation"" OR ""Tele rehabilitation"" OR ""Remote Rehabilitation"" OR ""Virtual Rehabilitation"" OR ""telemedicine"" OR ""telehealth"") AND (""smart devices"" OR ""device"" OR ""technology"" OR ""VR"" OR ""virtual reality"") AND (""hand"" OR ""upper extremity"" OR ""upper limb"")) | 57 |
| Google Scholar | ("stroke" OR "cerebrovascular accident" OR CVA)  ("telerehabilitation" OR telehealth OR telemedicine OR "remote rehabilitation")  ("smart device*" OR wearable* OR "virtual reality" OR VR) ("hand" OR "upper limb" OR "upper extremity") | 127 |
| Science Direct | (("Stroke" OR "Cerebrovascular Accident" OR "Apoplexy") AND ("Telerehabilitations" OR "Tele-rehabilitation" OR "Remote Rehabilitation") AND ("smart devices" OR "technology") AND ("upper extremity")) | 3 |
| Scopus | TITLE-ABS ( ( ( "Stroke" OR "Cerebrovascular Accident" OR "CVA (Cerebrovascular Accident)") AND ( "Telerehabilitations" OR "Tele-rehabilitation" OR "Tele rehabilitation" OR "Remote Rehabilitation" OR "Virtual Rehabilitation" OR "telemedicine" OR "remote care" OR "telehealth" ) AND ( "smart devices" OR "device" OR "technology" OR "VR" OR "virtual reality" ) AND ( "hand" OR "upper extremity" OR "upper limb" ) ) ) AND PUBYEAR > 2017 AND PUBYEAR < 2025 AND ( LIMIT-TO ( LANGUAGE , "English" ) ) AND ( LIMIT-TO ( DOCTYPE , "ar" ) OR LIMIT-TO ( DOCTYPE , "cp" ) ) | 50 |
| PubMed | ("stroke"[MeSH Terms] OR "stroke"[Title/Abstract] OR "cerebrovascular accident"[Title/Abstract] OR "CVA"[Title/Abstract]) AND ("telerehabilitation"[MeSH Terms] OR "telerehabilitation"[Title/Abstract] OR "remote rehabilitation"[Title/Abstract] OR "telehealth"[Title/Abstract]) AND ("smart devices"[Title/Abstract] OR "technology"[Title/Abstract] OR "virtual reality"[MeSH Terms] OR "virtual reality"[Title/Abstract] AND ("hand"[MeSH Terms] OR "hand"[Title/Abstract] OR "upper extremity"[Title/Abstract] OR "upper limb"[Title/Abstract]) | 36 |
| Web of Science | ((""Stroke"" OR ""Cerebrovascular Accident"" OR ""CVA (Cerebrovascular Accident"") AND (""Telerehabilitations"" OR ""Tele-rehabilitation"" OR ""Tele rehabilitation"" OR ""Remote Rehabilitation"" OR ""Virtual Rehabilitation"" OR ""telemedicine"" OR ""telehealth"") AND (""smart devices"" OR ""technology"" OR ""VR"" OR ""virtual reality"") AND (""hand"" OR ""upper extremity"" OR ""upper limb"")) | 190 |
